# Heterotopic ossification after total hip arthroplasty: When is development completed?

**DOI:** 10.1186/s13018-022-02959-z

**Published:** 2022-03-05

**Authors:** Roland E. Willburger, Friederike Brinkhoff, Jan Nottenkämper, Jan Krapp, Stella Oberberg

**Affiliations:** Department of Orthopaedic Surgery, Martin-Luther-Krankenhaus, Voedestrasse 79, 44866 Bochum, Germany

**Keywords:** Heterotopic ossification, Total hip arthroplasty, Brooker, Ossification prophylaxis

## Abstract

**Background:**

Heterotopic ossifications (HO) are a common complication after total hip arthroplasty (THA). Nonsteroidal anti-inflammatory drugs have proven to reduce the occurrence of HO. It is still unclear when the formation of HO is finished. Aim of our study was to answer this question.

**Methods:**

In a prospective study, the occurrence of periarticular HO was checked during the follow-up (FU) examinations. In total, 75 consecutive patients who underwent THA were included. To ensure a high follow-up rate, only patients with a life expectancy of at least 10 years were included. A medical ossification prophylaxis with mostly etoricoxib (90 mg once daily) was administered. Follow-up examinations were performed at 3 months, 1 year, 3, 5, and 10 years postoperatively. Each time, a clinical and radiological examination was carried out. The HO was graded according to Brooker’s method.

**Results:**

Low-grade HO classified by Brooker grade I and II occurred significantly more frequent than HO grade III. In patients with present HO, a possible increase in Brooker stage could further be observed within 3 years postoperatively. After 3 years, the formation of HO was completed in all patients.

**Conclusion:**

Three years after THA, the formation of HO is complete. After more than 3 years postoperatively, if HO occurs or increases, other triggering causes such as new trauma, periarticular infection, or implant loosening should be considered.

## Background

Heterotopic ossifications (HO) are a common complication after total hip arthroplasty (THA) [1]. The pathogenesis of HO has not yet been clarified with certainty. Several possible risk factors for the development of these HO are described in the literature [1–3]. Commonly mentioned risk factors are male gender, preexisting ossifications, and ankylosing spondylitis [1–3]. In addition, correlations between Brooker grade and ASA classification, age, and body mass index (BMI) have been described [4]. The surgical access routes to the hip joint are also discussed as having different levels of risk for the postoperative occurrence of HO [5, 6]. Complaints such as pain and limited joint mobility are described increasing with the amount of HO [7, 8]. Satisfaction rate of patients with HO is significantly lower compared to patients without HO [7]. In this context, only HO classified as Brooker grade III and IV seem to be clinically relevant [7]. Prophylactic therapy using radiation or drug prophylaxis with selective or non-selective antirheumatic drugs (NSAID) has proven to be effective in preventing HO [3, 9, 10]. The most common classification of HO is Brooker's classification, which divides the presence of HO in the radiograph of the hip in two planes into four grades of severity depending on its expansion and localization [11]. Many authors have shown that lower grades of HO are significantly more frequent than clinically relevant higher grades of HO in this context [1, 3–5, 7, 8, 10]. Most studies on HO refer to a follow-up (FU) period of 3 to 24 months [10]. Van Erp et al. (2021) investigated a period from 1 to 6 years postoperatively. This investigation still showed an increase in the rate of HO from 14 to 18% within the 5 years of the study period, regardless of Brooker classification [5]. However, as far as we know, there is no more differentiated consideration of the development of HO over several years, which describes at what time no more progression or new formation of HO may be expected. For this reason, this paper aims to show first the progression of HO over a period of 10 years with examinations after 3 months, 1 year, 3 years, 5 and 10 years. Special attention was paid to the individual development of HO as well as the appearance of new HO in the study period with the aim to provide information about the completion of the formation and development of HO.

## Materials and methods

In a prospective study, which was originally performed to investigate the long-term clinical and radiological outcomes and patient-related outcome measures (PROMs) of a collarless cementless hip stem (Polarstem, Smith & Nephew), we also assessed the occurrence of periarticular HO during the follow-up examination.

### Patients

In total, 75 consecutive patients who underwent primary cementless total hip arthroplasty (THA) in our department (between April 2009 and January 2010) were included. To ensure a high follow-up rate, only patients with a life expectancy of at least 10 years were included. Exclusion criteria (EC) for participation were general contraindications to the planned surgery such as systemic infections or a history of joint infection of the affected joint and the presence of severe comorbidities. In addition, severe back disease, neuropathic or neurosensory deficits, or massive bony insufficiency of the femoral or acetabular supporting apparatus of the affected hip were EC. Patients older than 75 years at the time of surgery were also excluded.

### Surgery and rehabilitation

The HI Cementless Threaded Cup System and the cementless Polarstem from Smith & Nephew were used in all patients. Depending on the individual requirements (like age), either a polyethylene ceramic or a polyethylene steel bearing pair with 28- or 32-mm heads was implanted. The operation was performed in a standardized procedure using a lateral approach modified according to Bauer in the supine position. Postoperatively, early mobilization was accompanied by physiotherapy on two forearm crutches under full load, avoiding forced external rotation, adduction, and flexion above 90° of the operated hip joints for 12 weeks. In all patients, drug therapy with predominantly 90 mg etoricoxib once daily (all but three patients who received 1800 mg ibuprofen or five mg prednisolone) was carried out postoperatively to reduce HO. The duration of application corresponds to the period required in the literature and proven to be sufficient [12]. After the hospital stay, all patients received either inpatient or outpatient follow-up treatment for at least 3 weeks.

### Clinical examination and questioning

All patients underwent standardized questioning preoperatively, 3 months, 1 year, 3 years, 5 years, and 10 years postoperatively according to the study protocol. This included a clinical examination with determination of range of motion using the neutral-zero method and assessment of the Harris Hip Score (HHS) and the hip osteoarthritis outcome score (HOOS). In addition, a two-plane X-ray of the hip joint was performed. Radiographs were evaluated for the presence and dimensions of periarticular ossifications using the Brooker classification, which is the established international method for radiographic classification and assessment of PAOs. The X-ray evaluation was carried out separately by the first and last author. If the radiographs were assessed differently, an experienced radiologist was called in and consensus was reached. All medical records were evaluated regarding the implementation and possible premature termination of drug ossification prophylaxis.

### Statistics

Data analysis was performed using the Microsoft Office Excel 2007 spreadsheet program. A descriptive evaluation (determination of frequencies) of the collected data was performed.

## Results

The mean age of the patient population at study inclusion was 66 years with a minimum of 37 and a maximum of 75 years. More women (64%) than men (36%) were included. Fifty-two patients (69%) received sufficient ossification prophylaxis postoperatively for at least 9 days. Twenty-three patients (31%) did not receive sufficient ossification prophylaxis. In three of these patients, the ossification prophylaxis was terminated prematurely due to questionable undesirable side effects (nausea (*n* = 1); elevation of transaminases (*n* = 1); diarrhea (*n* = 1)). In the remaining 20 patients, the prophylaxis was terminated prematurely for various reasons without undesirable side effects.

Three months postoperatively, two patients could not be followed up, one of them was followed up after 1, 3, and 5 years, the other only after 10 years (Brooker I). One year postoperatively, only one patient could not be followed up. This was the aforementioned patient who could only be re-examined after 10 years. Three years postoperatively seven patients could not be followed up (three had died regardless of the THA). Five years postoperatively 12 patients could not be re-examined (the aforementioned three had died regardless of the THA). Ten years postoperatively, 32 patients could not be followed up with X-rays (seven had died regardless of the THA, three were in poor general health and dementia, five could no longer be reached at the old address, six declined to re-examine but said that they had no problems with the THA, 11 patients or their relatives could only be interviewed by telephone). None of the patients followed had a serious complication in the area of the operated hip joint or revision surgery within 10 years.

Patients with adequate prophylaxis developed significantly less often HO and when HO occurred the extent was also significantly lower. None of the clinically relevant Brooker III or IV HO was detected in these patients.

**Fig. 1:**
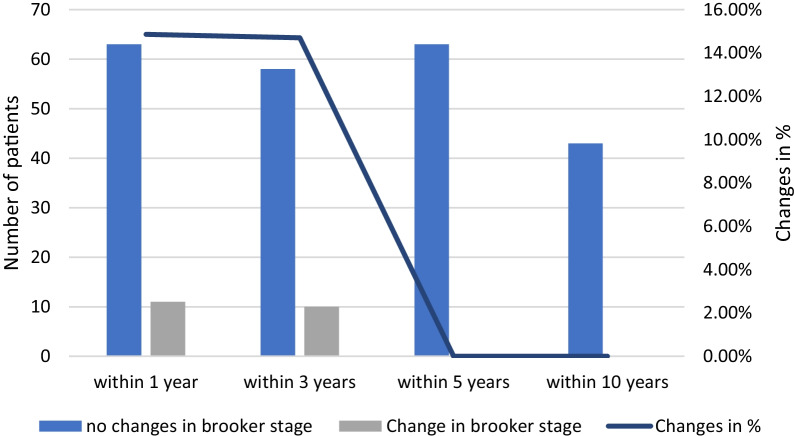
Changes in Brooker stage per patient for 10 years

In nine patients the expansion of the HO increased by one Brooker stage between the 1-year and 3-year follow-up examinations. When looking at each individual patient over a period of 10 years, no increase in Brooker’s stage was found in any patient after 3 years, regardless of whether or not adequate prophylaxis had been taken (Tables 1, 2; Fig. 1).

**Table 1:** HO prophylaxis and X-ray follow-up over 10 years

	Postop.	3 mon. FU	1 year FU	3 years FU	5 years FU	10 years FU
*N* =	75	73	74	68	63	43
Adequate HO prophylaxis	52 (69%)	50 (68%)	51 (69%)	46 (68%)	45 (71%)	31 (72%)
No/insufficient HO prophylaxis	23	23	23	22	18	12
X-rays	75	73	74	68	63	43
No X-rays	–	2 (3%)	1 (1%)	7 (9%)	12 (16%)	32 (43%)

**Table 2:** Brooker stages with and without sufficient HO prophylaxis over 10 years

	3 months FU		1 year FU		3 years FU		5 years FU		10 years FU	
*N* =	73		74		68		63		43	
	Proph.	no P.	Proph.	no P.	Proph.	no P.	Proph.	no P.	Proph.	no P.
Brooker 0	47 (64%)	14 (19%)	45 (61%)	9 (12%)	35 (51%)	6 (9%)	34 (54%)	5 (8%)	23 (53%)	3 (7%)
Brooker 1	2	7	5	9	10	8	10	6	7	5
Brooker 2	1	1	1	1	1	4	1	3	1	3
Brooker 3	0	1	0	4	0	4	0	4	0	1

## Discussion

Our study confirms that the prophylactic administration of cyclooxygenase-inhibitors (here mainly 90 mg etoricoxib once daily for 9 days) after the implantation of a primary cementless total hip replacement significantly reduces both the occurrence and severity of periarticular ossifications. Thus, the results of Winkler et al. [12] and Oberberg et al. [13] regarding the effectiveness of the cyclooxygenase-2-inhibitor etoricoxib to reduce HO are approved. In agreement with the literature, no higher-grade Brooker stages (III and IV) occurred in our patient collective after an adequately dosed and time-appropriate administration of the medical prophylaxis. The Brooker stages III and IV, which are generally described as clinically relevant, could be avoided.

Etoricoxib appeared to be an effective drug in reducing the rate and expression of HO. In our work, it was shown that after an adequate as well as a presumably too short drug prophylaxis, after more than 3 years postoperatively, no progression of HO could be determined. This observation is based on the comparison of native radiological images of the hip joint in 2 planes, without specifically detecting metabolic activity, for example, by scintigraphy. For medical ethical reasons, we did not routinely perform scintigraphy to assess metabolic activity in our study. However, indirectly, it can be concluded from our long-term study that HO activity can be regarded as probably completed after more than 3 years in the absence of an increase in expression on the native radiograph.

So far it hasn’t been studied when the formation of HO after primary THA is finished. However, this is relevant for determining the necessary follow-up time to check the effectiveness of various therapeutic methods for reducing HO. We are the first to show that 3 years after the implantation of a cementless total hip replacement, no further increase in HO is to be expected. If, after a period of more than 3 years after primary cementless THA HO occurs or increases, other triggering causes such as new trauma, periarticular infection, or implant loosening should be considered.

## Data Availability

All authors declare that all data and materials support the published claims and comply with field standards.

## Abbreviations

HO: Heterotopic ossification
THA: Total hip arthroplasty
HOOS: Hip osteoarthritis outcome score
HHS: Harris Hip Score
EC: Exclusion criteria
NSAID: Non-selective antirheumatic drugs (NSAID)
PROM: Patient-related outcome measures
FU: Follow-up
